# β‐Glucan produced by *Lentinus edodes* suppresses breast cancer progression via the inhibition of macrophage M2 polarization by integrating autophagy and inflammatory signals

**DOI:** 10.1002/iid3.876

**Published:** 2023-05-26

**Authors:** Fukai Zhu, Qianru Zhang, Jiexin Feng, Xiuru Zhang, Tingting Li, Shuwen Liu, Yanling Chen, Xiumin Li, Qici Wu, Yu Xue, Gulimiran Alitongbieke, Yutian Pan

**Affiliations:** ^1^ Engineering Technological Center of Mushroom Industry, School of Biological Science and Biotechnology Minnan Normal University Zhangzhou Fujian People's Republic of China; ^2^ Breast Surgery Department Zhangzhou Hospital of Fujian Medical University Zhangzhou Fujian People's Republic of China

**Keywords:** autophagic cell death, breast cancer, macrophage polarization, Nur77, β‐glucan from *Lentinus edodes* (LNT)

## Abstract

**Background:**

β‐Glucan from *Lentinus edodes* (LNT), an edible mushroom, possesses strong anticancer activity. However, the therapeutic effects of LNT during the occurrence and progression of breast cancer and their underlying molecular mechanisms have not been elucidated.

**Methods:**

*Mouse mammary tumor virus‐polyoma middle tumor‐antigen (MMTV‐PyMT)* transgenic mice were used as a breast cancer mouse model. Hematoxylin and eosin, immunohistochemical, and immunofluorescence staining were performed for histopathological analysis. Moreover, we developed an inflammatory cell model using tumor necrosis factor‐α (TNF‐α). Macrophage polarization was assessed using western blot analysis and immunofluorescence.

**Results:**

Orphan nuclear receptor 77 (*Nur77*) and sequestosome‐1 (*p62*) were highly expressed and positively correlated with each other in breast cancer tissues. LNT significantly inhibited tumor growth, ameliorated inflammatory cell infiltration, and induced tumor cell apoptosis in PyMT transgenic mice. Moreover, LNT attenuated the ability of tumors to metastasize to lung tissue. Mechanistically, LNT treatment restrained macrophage polarization from M1 to M2 phenotype and promoted autophagic cell death by inhibiting Nur77 expression, AKT/mTOR signaling, and inflammatory signals in breast tumor cells. However, LNT did not exhibit a direct pro‐autophagic effect on tumor cell death, except for its inhibitory effect on Nur77 expression. LNT‐mediated autophagic tumor cell death depends on M1 macrophage polarization. In in vitro experiments, LNT inhibited the upregulation of p62, autophagy activation, and inflammatory signaling pathways in Nur77 cells.

**Conclusion:**

LNT inhibited macrophage M2 polarization and subsequently blocked the AKT/mTOR and inflammatory signaling axes in breast cancer cells, thereby promoting autophagic tumor cell death. Thus, LNT may be a promising therapeutic strategy for breast cancer.

## Graphical abstract

*Lentinus edodes* (LNT) inhibited macrophage M2 polarization, and subsequently blocked the AKT/mTOR and inflammatory signaling axis in breast cancer cells, thereby promoting autophagic tumor cell death. Possibly, LNT may be a promising therapeutic strategy for breast cancer.

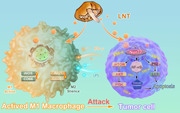

## 1 INTRODUCTION

Breast cancer is one of the most common malignancies and a serious threat to women health worldwide.^1^ Endocrine therapy is a first‐class, target‐directed therapy approved for the treatment of breast cancer. However, only 20%–40% of patients with advanced estrogen receptor (ER)^+^ breast cancer benefit from endocrine therapy because of acquired resistance against the therapy by other patients.^2^ Moreover, the 5‐year survival rate of patients with metastatic breast cancer is less than 30% after adjuvant therapy.^3^ Therefore, more effective and nontoxic therapeutic strategies based on natural products with anticancer properties are being explored.^4^ Hence, the discovery and development of anticancer drugs from natural resources will become a focus of anticancer drug intervention studies.

Tumor‐associated macrophages (TAMs), vital components of the tumor microenvironment (TME), are involved in tumorigenicity, including proliferation, invasion, and metastasis, of cells.^5^, ^6^, ^7^ TAMs are not polarized until they receive specific microenvironmental signals.^8^ The alternative polarization of macrophages, that is, M1‐like or M2‐like, can be induced by various stimuli from tumor cells or other cells in the TME.^9^ M2‐like macrophages secrete oncogenic cytokines, growth factors, and proteases, which increase their tumorigenicity.^10^ M2 polarization of TAMs, a major factor in breast cancer malignancy and metastasis, can be induced in breast cancer cells.^11^ The oncogene T‐cell malignancy 1‐stimulates interleukin (IL)‐6 secretion in triple‐negative breast cancer cells, which promotes M2 polarization of macrophages to increase invasiveness.^12^ Moreover, the accumulation of hyaluronan facilitates macrophage infiltration into breast cancer tissues, and its fragments can enhance macrophage differentiation into M2‐like macrophages.^13^ Therefore, the interaction between polarized macrophages and breast cancer cells is involved in breast cancer progression.

Lentinan (LNT), a β‐glucan produced by *Lentinus edodes*, contains two β‐1,6‐glucose branches; *L. edodes* was the first medicinal fungus to be used in the field of modern biotechnology. LNT possesses antitumor activity because of its unique triple‐helical conformation.^14^ Several studies have reported that LNT alone or in combination with other chemotherapeutic agents can be used for the treatment of lung,^15^ gastric,^16^ hepatic,^17^ and ovarian cancers.^18^ A previous study reported the effect of LNT extract on breast cancer therapy.^19^ LNT is clinically used as an antitumor agent.^20^, ^21^ As an immunomodulator, LNT stimulates natural killer cell activity,^22^, ^23^ macrophage/monocyte function (secretion of IL‐1 and superoxide anions), phagocytosis, and cytotoxicity.^24^, ^25^, ^26^ However, whether LNT affects breast cancer progression involving crosstalk between cancer cells and macrophage polarization remains unclear.

In the present study, we investigated whether LNT exerts its anticancer effects by targeting inflammation and macrophage polarization. We also investigated the mechanisms underlying the regulation of macrophage polarization and autophagic cell death by LNT in breast cancer cells. This study will provide novel insights into the underlying mechanisms of LNT‐induced tumor suppression and indicate whether β‐glucan or other foods containing β‐glucan could be used for treating patients with lung metastasis of breast cancer.

## 2 MATERIALS AND METHODS

### 2.1 Tissue specimens and patients

This study was approved by the Ethics Committee of Minnan Normal University. We collected 111 clinical breast cancer samples and paired adjacent tissue samples between November 2013 and January 2016 from the Zhongshan Hospital of Xiamen University. The clinical data of the enrolled patients are presented in Table 1. All participants and researchers signed an informed consent form. The inclusion criteria were as follows: female patients aged 18–70 years who were diagnosed with breast cancer based on pathology, and none of the patients received chemotherapy or radiotherapy before surgery. The exclusion criteria were as follows: participation in other clinical trials, pregnancy and lactation, failure to sign the written informed consent form, and history of alcohol or drug abuse.

**Table 1:** Clinical characteristics of patients.

Parameters	Number of patients	Intrinsic subtypes of breast cancer				*p* Value
		Luminal A	Luminal B	HER2 positive	Triple negative	
Number	111	27	60	20	4	
Age						
<Mean (47)	76	19	39	15	3	.105
≥Mean (47)	35	8	21	5	1	
Tumor size						
T1–2	109	26	59	20	4	.700
T3–4	2	1	1	0	0	
Nodal status						
N0–1	86	19	52	11	4	.456
N2–3	25	8	8	9	0	
Metastasis						
M0	47	12	24	8	3	**.016** *
M1	64	15	36	12	1	
Pathological stage						
I–II	81	17	47	13	4	.292
III–IV	30	10	13	7	0	

*Note*: Bold value indicates the significant difference *p* < 0.05.

* *p* < 0.05.

### 2.2 Cell culture and cell treatment conditions

Human breast cancer cell line T47D and Raw264.7 macrophage cell line were obtained from the American Type Culture Collection of the Chinese Academy of Sciences. All cells were cultured in high‐glucose Dulbecco's modified Eagle's medium (DMEM) (Gibco) supplemented with 1% penicillin/streptomycin and 10% (v/v) (Gibco) fetal bovine serum (FBS) (Gibco) and maintained at 37°C in a 5% CO_2_ incubator (Thermo Fisher Scientific). The cells were seeded in 6‐well or 24‐well plates at a density of 10^5^ cells/cm^2^ a day before the experiments. Tumor necrosis factor‐α (TNF‐α) (20 ng/mL) and different concentrations of LNT (50, 125, 250, 500, and 1000 μg/mL) were added to the cells at 70%–80% confluence, and the plates were incubated for 24 h.^27^ Conditioned medium was prepared by culturing tumor cells in medium supplemented with β‐glucans or phosphate buffer (PBS) for 24 h, and the supernatant was used as the medium for culturing the macrophages; after 24 h of incubation in the conditioned medium, the supernatant of the macrophage culture was used to treat T47D tumor cells.

### 2.3 Animal experiments

Specific pathogen‐free (SPF) mouse mammary tumor virus‐polyoma middle tumor‐antigen (MMTV‐PyMT) transgenic mice (strain type: friend virus B‐type, FVB), which is a common breast cancer mouse model, were purchased from The Jackson Laboratory. The animals were fed normal mouse food and water ad libitum under SPF conditions and a fixed temperature of 22 ± 2°C, 12‐h dark‐light cycle, and relative humidity of 40%–60%. All animal experiments were approved by the Animal Ethics Committee of Minnan Normal University (AEWC‐2021010). The genotype of 2‐week‐old was identified using the following primer sequence: forward: 5′‐ GGAAGCAAGTACTTCACAAGGG‐3′; reverse: 5′‐GGAAAGTCACTAGGAGCAGGG‐3′). Before the experiments, all mice were fed under SPF conditions for at least 1 week. We selected 4‐week‐old female MMTV‐PyMT transgenic mice with similar initial body weight and growth status and randomly divided them into the following three groups: control and 1 and 20 mg/kg LNT solution‐treated groups (six mice per group). Each mice in the drug‐treated group was intravenously administered 1 or 20 mg/kg LNT solution via the tail vein for 6 weeks. The control group was administered 0.9% normal saline via the tail vein. Three hours after the last drug administration, all mice were killed by cervical dislocation, and the breast tumor, heart, liver, spleen, kidney, and lung tissues were immediately dissected, photographed, and weighed. Subsequently, the tumor and lung tissue samples were used for protein extraction, immunohistochemistry, and immunofluorescence staining.

### 2.4 Quantitative real‐time polymerase chain reaction (qPCR)

The expression levels of the Orphan nuclear receptor 77 gene (*Nur77*) and sequestosome‐1 gene (*p62I)* in human breast tumor tissue samples were determined using qPCR. RNA was extracted using the TRIzol reagent (Ambion) according to the manufacturer's instructions, and 1–2 μg of total RNA was used for reverse transcription using the SuperScript III First‐Strand Synthesis Kit (Invitrogen) and oligo‐dT priming as per the manufacturer's instructions. SYBR Premix Ex Taq (Takara) was used to perform qPCR according to the manufacturer's cycling conditions (40 cycles) in a Bio‐Rad C1000 Thermal Cycler. The following primers were used to determine relative gene expression: *Nur77*: forward, 5′‐GGCATGGTGAAGGAAGTTGT‐3′; reverse, 5′‐CAGGGAAGTGAGGAGATTGG‐3′; and *p62*: forward, 5′‐ATCGGAGGATCCGAGTGT‐3′; reverse, 5′‐TGGCTGTGAGCTGCTCTT‐3′. Data were analyzed using the $2^{‐∆∆C_{t}}$ method.^28^

### 2.5 Western blot analysis

The tumor tissue samples were cut into small pieces, and the pieces and T47D cells were incubated in ice for 20 min in RIPA lysis buffer (Beyotime). After centrifugation at 14,000 rpm for 5 min, the supernatant was collected and used for protein expression analysis. Equal amounts of proteins were electrophoresed on a sodium lauryl sulfate‐polyacrylamide gel; the proteins were transferred to a polyvinylidene fluoride (PVDF) membrane (Beyotime) and blocked using 5% skim milk powder (Beyotime) for 1 h. Then appropriately diluted primary antibodies against Nur77 (12235AD; Proteintech), p62 (AF5384; Affinity), LC3B (ab51520; Abcam), P‐mTOR (AF3308; Affinity), P‐AKT (AF0239; Affinity), phosphorylated IkappaB kinase (P‐IKK) (AF3013; Affinity), IkappaB alpha (IκBα) (AF5002; Affinity), proliferating cell nuclear antigen (PCNA) (AF0239; Affinity), cleaved caspase‐3 (AF7022; Affinity), reduced glyceraldehyde‐phosphate dehydrogenase (GAPDH) (T0004; Affinity), β‐actin (T0022; Affinity), PARP1 (DF7198; Affinity), and LC3 (ARG55622; Arigobio) were added, and the membrane was incubated at 4°C overnight. After washing the membrane, the corresponding secondary antibody (Beyotime) was added, and the membrane was incubated for 1 h. Chemiluminescence was detected using an enhanced chemiluminescence reagent (Beyotime). After development and fixing, the membrane was photographed using a gel imaging analysis system (UniCel DxI800; Beckman Coulter).

### 2.6 Immunohistochemical analysis

Immunohistochemical analysis was performed as previously described.^29^ Briefly, the solid tumors were removed, fixed using 10% formaldehyde, and embedded in paraffin. The sections were blocked using 0.3% hydrogen peroxide at room temperature for 20 min to remove endogenous peroxidase activity. Next, nonspecific protein binding was blocked by incubation the sections with 2% bovine serum albumin and with primary antibody against Nur77 (12235AD; Proteintech), p62 (AF5384; Affinity), CD68 (ab955; Abcam), and Ki67 (ab15580; Abcam) overnight at 4°C; then, the secondary antibody was added, carried with horseradish peroxidase, and developed according to the manufacturer's protocol (IHC staining module; Beijing Zhongshan Biotechnology). The sections were counterstained using hematoxylin, and the immunohistochemical staining results were observed under a light microscope (Olympus IX71).

### 2.7 Immunofluorescence staining

Frozen sections were subjected to immunofluorescence staining using primary antibodies against CD86 (diluted 1:100; Sigma‐Aldrich), CD206 (monoclonal; diluted 1:100; Invitrogen), and PCNA (AF0239; Affinity) at 4°C overnight. On the 2nd day, the slides were washed thrice using 1× PBS for 5 min each time and incubated with the corresponding secondary antibody (diluted 1:500; Molecular Probes) for 1 h at room temperature. Diamidinophenyl indole (DAPI) (diluted 1:300; Beyotime) was used to visualize the nuclei. CD86‐ and CD206‐positive cells were detected using a confocal laser‐scanning microscope (Leica DIM8).

### 2.8 Hematoxylin and eosin (H&E) staining

Mouse breast tumor and lung tissue samples were cut and immersed in 10% formalin at 4°C for >24 h. The tissue samples were dehydrated using graded ethanol solution (2 h for each concentration: 80%, 90%, 95%, and 100%), immersed in xylene (10 min/3 times), and embedded in paraffin. The embedded tissues were cut into 5‐μm sections. Following dewaxing using xylene (10 min/3 times) and hydration using graded ethanol (5 min for each concentration: 100%, 100%, 95%, 90%, 80%, and 70%), the sections were stained using H&E (Beyotime) according to the method reported in a previous study.^30^ Each sample group was observed under an optical microscope (Olympus IX71).

### 2.9 Terminal deoxynucleotidyl transferase‐mediated dUTP nick‐end labeling (TUNEL) assay

Apoptosis of tumor cells in the control and LNT‐treated groups was investigated using a one‐step terminal deoxynucleotidyl transferase mediated dUTP nick‐end labeling (TUNEL) apoptosis assay kit (Beyotime). Briefly, PBS (Beyotime) containing 0.5% Triton X‐100 (Beyotime) was added to the tissue sample slides and incubated for 5 min at room temperature. Next, TUNEL test solution was prepared according to the manufacturer's instructions and fully mixed, TUNEL test solution (50 μL) was added to the slides, and the slides were incubated at 37°C for 60 min in the dark. Finally, DAPI staining was performed to visualize the nuclei. After blocking using an anti‐fluorescence quenching mounting solution, the apoptotic cells were observed under a fluorescence microscope (Leica DIM8).

### 2.10 Statistical analysis

Data are presented as mean ± standard deviation (SD) and analyzed using the SPSS 25.0 software (SPSS Inc.). H&E, immunohistochemistry, and immunofluorescence staining results were analyzed using the Image J software. Quantitative analysis was performed using the Quantity One software. Data were statistically analyzed using one‐tailed Student's *t* test or one‐way analysis of variance (ANOVA) using the GraphPad Prism 7.0. Statistical significance was set at *p* < .05.

## 3 RESULTS

### 3.1 Nur77 and p62 are highly expressed and positively correlated with each other in breast cancer tissues

**Figure 1:**
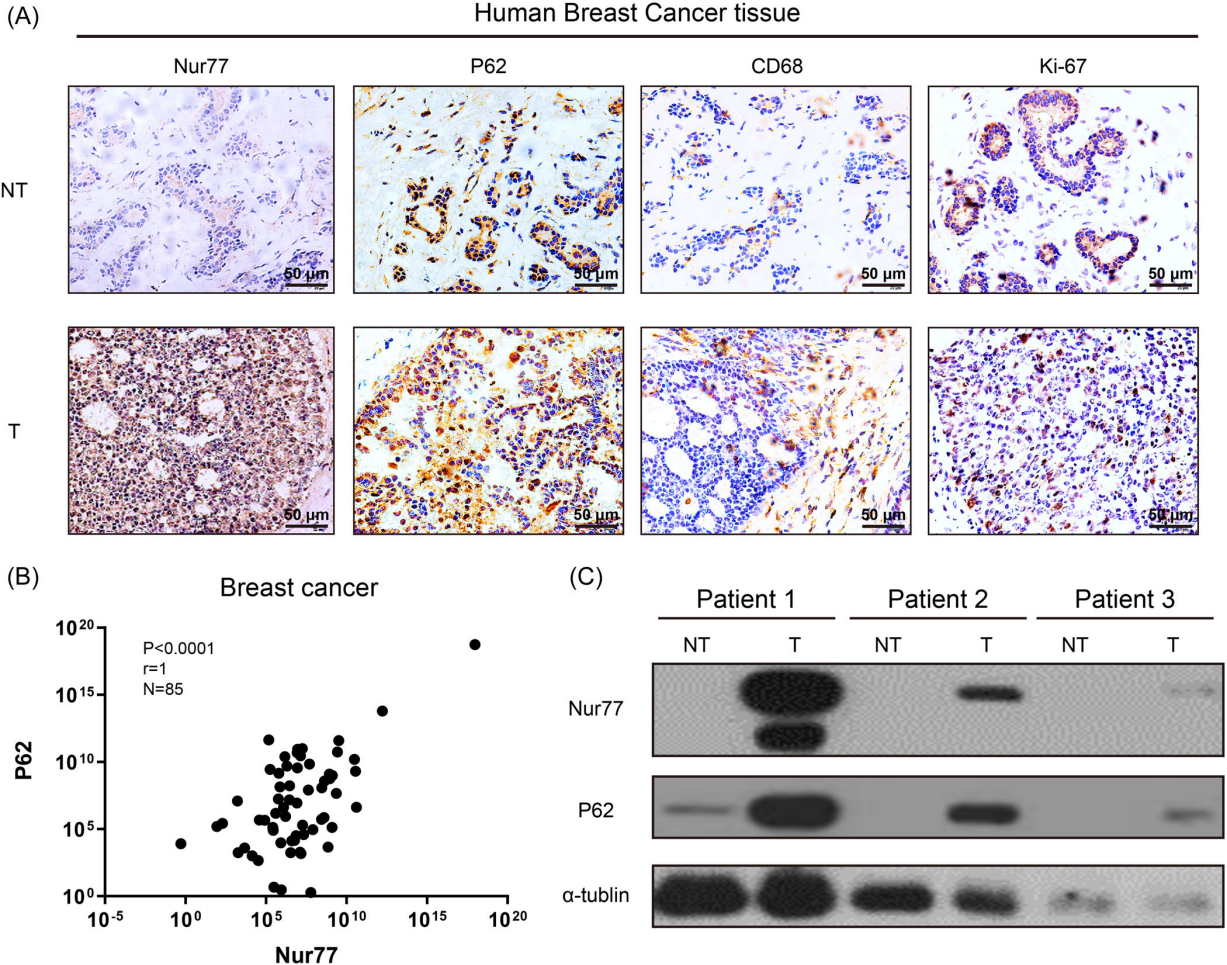
Expression of Nur77 and p62 in human breast cancer tissue samples. (A) Expression of Nur77, p62, CD68, and Ki‐67 proteins in T and NT tissue samples as per the immunohistochemical analysis. Scale bar: 50 μm. (B) mRNA levels of Nur77 and p62 in T and NT tissue samples as per qPCR. (C) Expression of Nur77 and p62 proteins in T and NT tissue samples as per western blot analysis analysis (*n* = 3). Data are shown as mean ± SD, *n* ≥ 3. *p < .05; **p < .01. NT, nontumor; qPCR, quantitative real‐time polymerase chain reaction; T, tumor.

Immunohistochemical analysis revealed that the number of Ki67‐positive cells was significantly increased in tumor tissues compared to that in nontumor tissues. Moreover, CD68 expression was increased tumor tissues compared to that in nontumor tissues, suggesting that macrophages aggregated in the breast tumor tissues (Figure 1A). Moreover, the expression of Nur77 and the autophagy‐related molecule p62 increased in tumor tissues compared to that in nontumor tissues (Figure 1A). Furthermore, the qPCR results elucidated a positive association between *Nur77* and *p62* in breast tumor tissues (Figure 1B). Moreover, breast tumors with high Nur77 levels also exhibited high p62 expression (Figure 1C). These results indicated that Nur77 and p62 are highly expressed and positively correlated with each other in cancer tissues.

### 3.2 LNT inhibits breast cancer growth in PyMT transgenic mice

**Figure 2:**
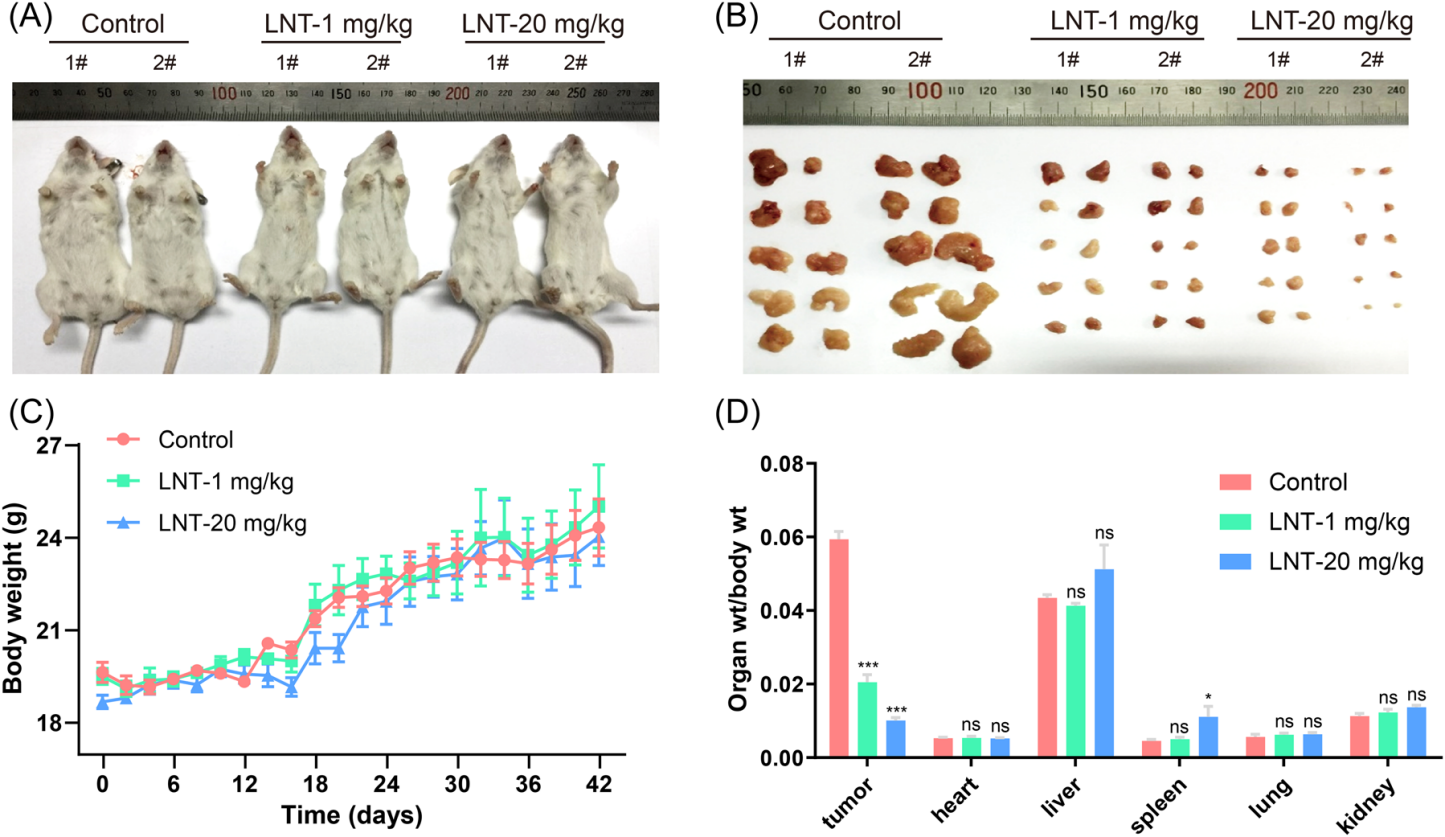
Effects of β‐glucan from *Lentinus edodes* (LNT) on tumor growth in PyMT transgenic breast cancer mouse model. (A) Images of tumor‐bearing PyMT transgenic mice; the three groups of mice were as follows: Control group, LNT (1 mg/kg)‐treated group, and LNT (20 mg/kg)‐treated group. (B) Mammary tumor size in the LNT‐treated and control groups after modeling. (C) Body weight statistics for LNT‐treated and control groups. (D) Ratio of organ weight to body weight (tumor, heart, liver, spleen, lung, and kidney) in LNT‐treated and control mice. Data are shown as mean ± SD, *n* ≥ 3.

Some medicinal mushrooms exhibit anticancer activities such as inhibition of breast cancer growth and lung metastasis.^31^ Here, we investigated the preventive effects of LNT on the occurrence of breast cancer in MMTV‐PyMT transgenic mice. Genotypic identification of the MMTV‐PyMT transgenic mice was performed using RT‐PCR (Supporting Information: Figure S1). In the control group, obvious breast tumor contour features were observed at the age of 3 months (10‐week‐old), but breast tumors did not appear in the LNT‐administered mice. After 6 weeks of LNT administration, the tumor size in each pair of 10 mammary glands was inhibited in the LNT‐treated group compared to that in the control group, and the inhibitory effect increased with increasing drug concentration (Figure 2A,B). However, the body weight of the mice from the LNT‐treated group was not significantly different from that of the mice of the control group (Figure 2C). Moreover, the ratio of organ weight to body weight, including the tumor, heart, liver, spleen, lung, and kidney, did not exhibit any changes among the three groups (Figure 2D). These data indicated that LNT administration inhibited breast tumor growth in PyMT transgenic mice.

### 3.3 LNT promotes cell apoptosis in breast tumor tissues via the regulation of autophagic cell death and inflammatory signaling

**Figure 3:**
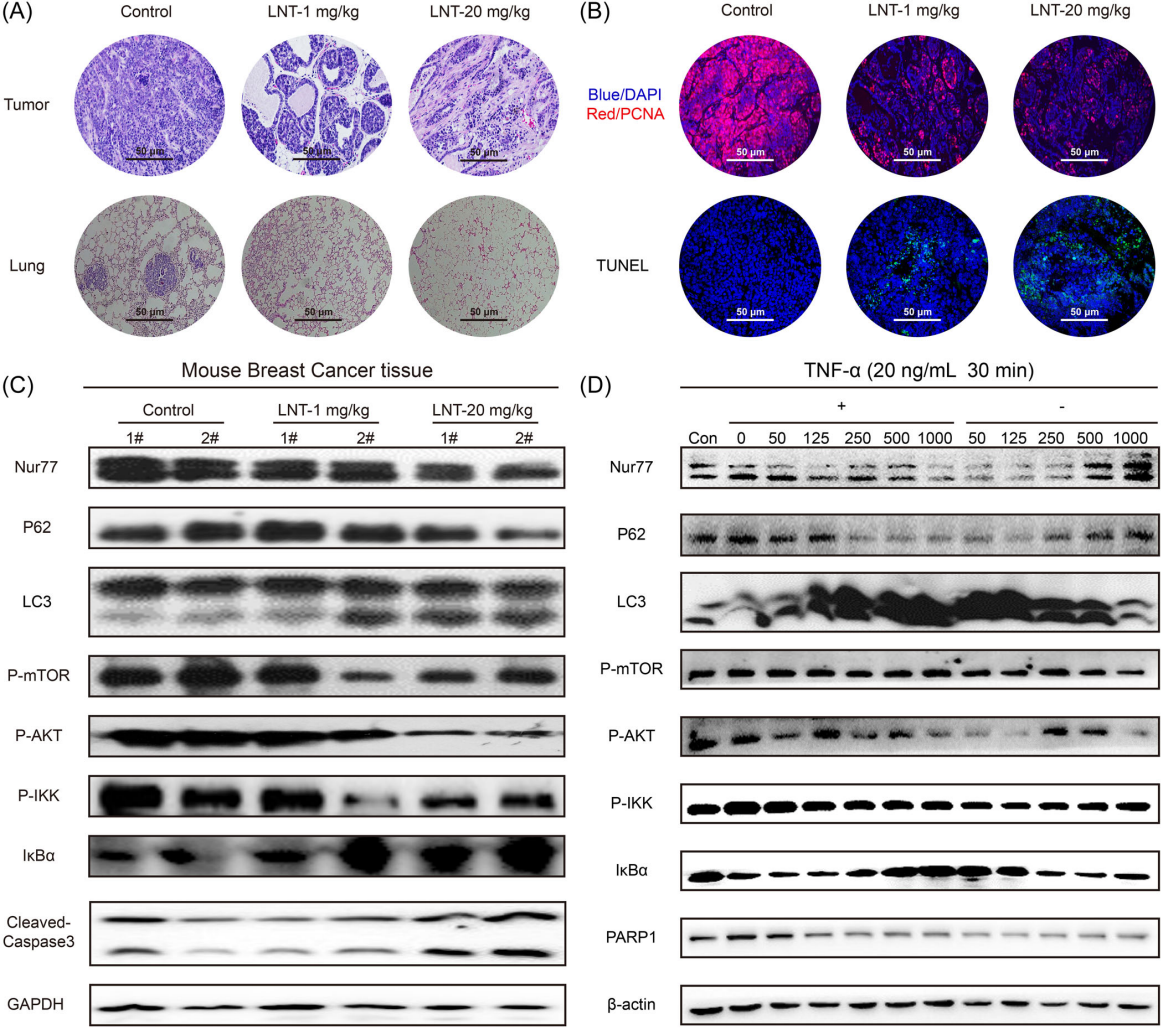
Effect of *Lentinus edodes* (LNT) on cell proliferation and cell apoptosis in tumor tissues and breast cancer cell lines. (A) Hematoxylin and eosin (H&E) staining (the top images) was used to investigate inflammation and infiltration in the LNT‐treated group and the control group and for the examination of lung metastasis (lower images) of LNT‐treated group and control group. (B) Immunofluorescence staining was used to detect the expression of proliferation and apoptosis‐associated proteins using antibodies against PCNA in the LNT‐treated and control groups (upper images), and TUNEL assay was performed to assess cell apoptosis in the tumor tissue of LNT‐treated and control groups (lower images). (C) Expression of Nur77, p62, LC3, p‐mTOR, p‐AKT, p‐IKK, IKBα, PCNA, cleaved caspase‐3, and GAPDH proteins in tumor tissues as per western blot analysis analysis. (D) Expression of Nur77, p62, LC3, p‐mTOR, p‐AKT, p‐IKK, IKBα, PARP1, and β‐actin proteins in tumor cells as per western blot analysis analysis. Data are shown as mean ± SD, *n* ≥ 3.

To further explore the LNT‐mediated antitumor effect, we investigated proliferation and apoptotic events in tumor tissues. H&E staining revealed that the number of inflammatory cells in the LNT group was lower than that in the control group (Figure 3A), indicating that LNT inhibited the infiltration of immune cells in tumor tissues in a dose‐dependent manner (Figure 3A). Moreover, LNT treatment suppressed lung metastasis of cancer cells compared to that in control mice (Figure 3A). Compared to that in the control group, PCNA expression decreased in the mammary gland tumor tissues from the LNT‐administered mice (Figure 3B). Additionally, LNT injection enhanced apoptotic signals in tumor tissues compared to those in the control tumors (Figure 3B, bottom). Next, we verified the mechanism underlying the inhibition of breast cancer by LNT. After LNT treatment, Nur77 expression gradually decreased with an increase in drug dose (Figure 3C and Supporting Information: S2A). We also observed an increase in the expression of apoptotic marker cleaved caspase‐3 and a decrease in PCNA and p‐AKT expression in LNT‐treated cells, indicating the antiproliferative and proapoptotic effects of LNT (Figure 3C and Supporting Information: S2A). Moreover, LNT treatment increased the LC‐3II/LC3I ratio and decreased p62 and p‐mTOR levels, suggesting autophagic activation in LNT‐administered tumors. LNT also induced the expression of inflammation‐related molecule IKBα and decreased that of p‐IKK, suggesting the anti‐inflammation effect of LNT (Figure 3C and Supporting Information: S2A). The results revealed that LNT promoted autophagic cell death in breast cancer cells by inhibiting the expression of AKT/mTOR and nuclear factor‐kappaB (NF‐κB) signaling pathway‐related proteins (Figure 3C and Supporting Information: S2A). The in vitro experiment elucidated that after treatment of T47D cells with TNF‐α in combination with LNT, a concentration‐dependent downregulation of Nur77, AKT/mTOR, and NF‐κB signaling pathway‐related proteins and promotion of autophagic cell death was observed compared to that in the cells treated with LNT alone (Figure 3D and Supporting Information: S2B). Therefore, LNT significantly inhibited tumor cell proliferation and promoted autophagic death, thereby inhibiting breast cancer progression.

### 3.4 LNT exerts antitumor effects by inhibiting M2 macrophage polarization

**Figure 4:**
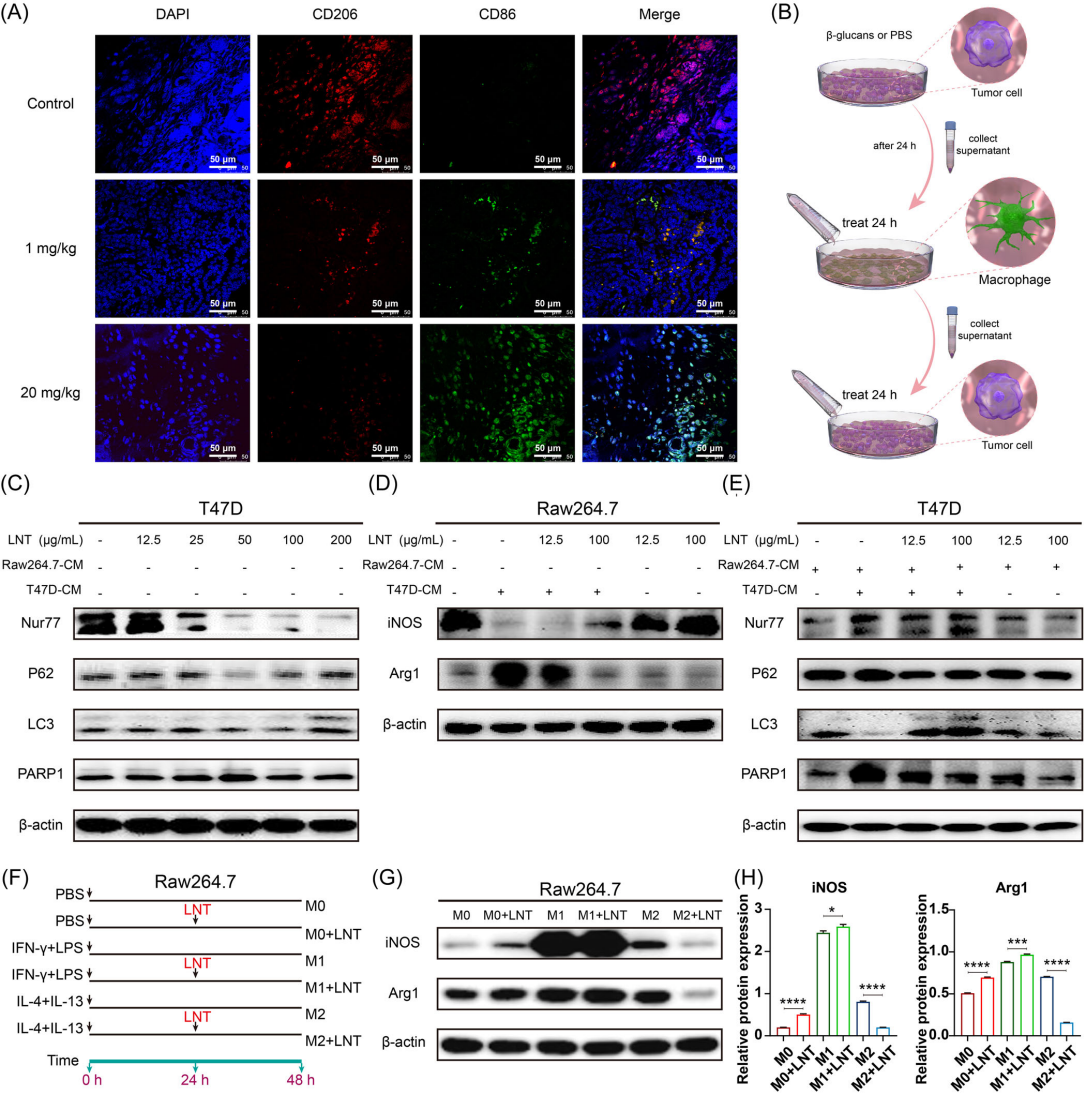
Macrophage polarization in tumor tissues and tumor cells. (A) Immunofluorescence analysis was used to detect the number of CD206‐ and CD86‐positive cells in the *Lentinus edodes* (LNT)‐treated and control groups; CD206‐ (red) and CD86‐positive (green) cells indicate M2 and M1 macrophages, respectively. (B) Schematic diagram of cell supernatant transfer culture. (C) T47D cells were treated with LNT, and the expression of Nur77, p62, LC3, PARP1, and β‐actin proteins was analyzed in different groups using western blot analysis. (D) Macrophages treated with culture medium from LNT‐treated T47D cells was used to treat macrophages, and the expression of iNOS, Arg1, and β‐actin proteins in different groups was analyzed using western blot analysis. (E) T47D cells treated with culture medium from macrophages (pretreated with medium of LNT‐treated T47D cells); expression of Nur77, p62, LC3, PARP1, and β‐actin proteins in different groups as per western blot analysis analysis. (F) Schematic diagram of macrophage‐directed differentiation culture induced by cytokines with different properties. (G) Expression of iNOS, Arg1, and β‐actin proteins in different groups as per western blot analysis analysis. (H) Semiquantitative analysis of iNOS and Arg1 proteins in panel (G). Data are shown as mean ± SD, *n* ≥ 3. *p < .05; **p < .01; ***p < .001; ****p < .0001.

We investigated the phenotype of macrophages to explore the specific regulatory mechanism of LNT in the occurrence of breast cancer. We observed that the expression of the M1 macrophage marker CD86 significantly increased, whereas that of the M2 macrophage marker CD206 significantly decreased after treatment with different concentrations of LNT (Figure 4A). For cell line experiments, we designed the following two cell culture methods: cell supernatant transfer culture (Figure 4B) and macrophage‐directed differentiation culture induced by cytokines with different properties (Figure 4F). The results revealed that different concentrations of LNT did not significantly affect the expression of autophagy‐related or apoptotic proteins, but Nur77 expression was significantly inhibited in T47D cells (Figure 4C). After treatment with the supernatant of T47D cells pretreated with different concentrations of LNT, the macrophages exhibited obvious directional differentiation. The supernatant from the untreated tumor cells significantly induced the expression of the M2 macrophage‐related marker arginase 1 (Arg1) and inhibited that of the M1 macrophage‐related marker inducible nitric‐oxide synthase (iNOS) (Figure 4D, second row). However, the medium from LNT‐treated cancer cells induced an increase in iNOS expression and a decrease in that of Arg1 in a dose‐dependent manner (Figure 4D, third and fourth rows). However, iNOS and Arg1 expression did not change in LNT‐treated macrophages (not conditioned medium) compared to that in the control cells (Figure 4D, fifth and sixth rows vs. first row). Therefore, LNT‐mediated macrophage M1 polarization depends on the crosstalk between tumor cells and macrophages.

Additionally, the macrophage culture medium (pretreated with the medium from T47D cells) inhibited LC3II/LC3I expression and induced that of full‐length PARP1 and p62, suggesting that autophagy was activated (Figure 4E, second row vs. first row). However, the macrophage culture medium (pretreated with the LNT‐exposed medium of T47D cells) induced LC3II/LC3I expression and inhibited that of p62 and full‐length PARP1 (Figure 4E, third and fourth rows vs. second row). In contrast, the macrophage culture medium (pretreated with LNT) did not affect the autophagic flux and apoptosis of breast cancer cells (Figure 4E, fifth and sixth rows vs. first row). Moreover, the macrophage culture medium (pretreated with the medium from T47D cells or pretreated with LNT‐exposed medium from T47D cells) significantly promoted Nur77 expression; however, the medium (pretreated with LNT directly) did not affect Nur77 expression (Figure 4E). These data establish that the crosstalk between macrophages and tumor cells subsequently promoted autophagic cell death in breast cancer cells, which may depend on Nur77 expression.

To confirm the effects of LNT on macrophage polarization, we used cytokines with different properties to induce directional macrophage differentiation (Figure 4F). Western blot analysis analysis revealed that Arg1 was significantly downregulated in LNT‐treated M2 macrophages, whereas Arg1 expression did not change in either M1 macrophages or LNT‐treated M1 macrophages (Figure 4G,H). However, iNOS was significantly upregulated in LNT‐treated M1 macrophages, and its expression was inhibited in LNT‐treated M2 macrophages (Figure 4G,H). These data further demonstrated that LNT could regulate the phenotype of macrophages in the TME and affect their directional differentiation.

### 3.5 LNT blocks TNF‐α‐induced autophagic cell death of breast cancer cells in a Nur77‐dependent manner

**Figure 5:**
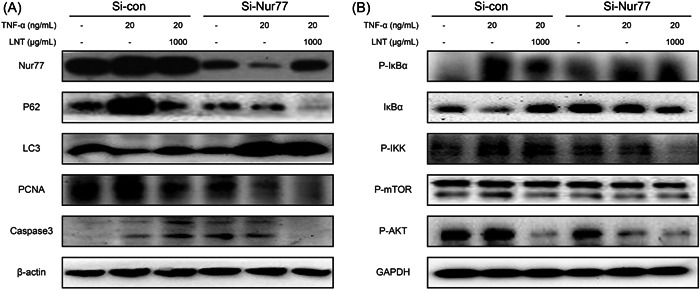
Effects of LNT on TNF‐α‐mediated autophagic cell death in breast cancer cells. T47D cells were transfected with Nur77 siRNA and untransfected cells were used as control. The cells were treated with 20 ng/mL TNF‐α alone or a combination of TNF‐α and 1000 μg/mL LNT. (A) Expression of Nur77, p62, LC3, PCNA, caspase‐3, and β‐actin proteins in different groups as per western blot analysis analysis. (B) Expression of pIKBα, IKBα, p‐IKK, p‐mTOR, p‐AKT, and GAPDH proteins in different groups as per western blot analysis analysis. Data are shown as mean ± SD, *n* ≥ 3. LNT, *Lentinus edodes*; TNF‐α, tumor necrosis factor‐α.

To confirm LNT‐mediated anticancer effects and autophagic cell death via Nur77, we silenced *Nur77* in T47D cells. After TNF‐α treatment, the expression of Nur77, p62, PCNA, p‐IκBα, p‐IKK, p‐AKT, and p‐mTOR increased and that of caspase‐3 and total IκBα decreased, suggesting that TNF‐α inhibited autophagic cell death and the NF‐κB inflammatory signaling pathway (Figure 5A,B, second row vs. first row; Supporting Information: Figure S3A,B). However, LNT administration significantly reversed the changes induced by TNF‐α treatment, indicating that LNT could inhibit the effects of TNF‐α on autophagic cell death (Figure 5A,B, third row vs. second row; Supporting Information: Figure S3A,B). *Nur77* silencing inhibited the autophagic cell death and NF‐κB inflammatory signaling pathways that were mediated by TNF‐α. The TNF‐α and LNT combination more significantly inhibited autophagic cell death (Figure 5A,B and Supporting Information: Figure S3A,B). Therefore, the effect of LNT on p62/mTOR, NF‐κB signaling pathway, and autophagic cell death on tumor cells stimulated by TNF‐α could also depend on Nur77.

## 4 DISCUSSION

Glucans belong to a group of polysaccharides found in the cell wall of bacteria, fungi including mushrooms, and cereals, such as barley and oats.^32^ They are considered biological response modifiers with immunomodulatory and health‐beneficial effects, including anticancer properties.^32^ LNT interferes with the metastasis of colon cancer or B16‐BL6 melanoma cells to the lungs in a dose‐dependent manner,^33^ and it activates immune responses to suppress cell proliferation and induce cell apoptosis.^34^, ^35^ Moreover, LNT suppresses cell proliferation and promotes apoptosis in ER^+^ breast cancer.^36^ Similarly, we observed that LNT‐induced breast cancer cells secreted unique extracellular compounds to stimulate the directed differentiation of macrophages, thereby promoting autophagic cell death. To the best of our knowledge, this study is the first to elucidate the interaction between tumor cells and macrophages during the progression of LNT‐mediated antitumor effects.

As an early response gene, *Nur77* (also known as *TR3*) regulates many key life processes, such as cell proliferation, apoptosis, embryonic development, and angiogenesis.^37^ Qin et al. reported that Nur77 promotes cigarette smoke‐induced autophagic cell death by increasing the dissociation of Bcl2 from beclin‐1.^38^ Abnormal expression or function of Nur77 can also lead to various diseases, including breast cancer.^27^, ^39^, ^40^ Previous studies have reported that p62 plays a role in inhibiting cell autophagy signals; affects proliferation, differentiation, and apoptotic events; is abnormally expressed as an oncogenic factor in several types of tumors; and inhibits the mTOR signaling pathway to coregulate tumorigenesis.^41^, ^42^ TRIM59 promotes breast cancer motility by suppressing p62‐selective autophagic degradation of PDCD10,^43^ and SH003 suppresses breast cancer growth by accumulating p62 in autolysosomes. Furthermore, the composite multivalent interaction between Nur77 and p62 coordinates autophagy by sequestering damaged mitochondria and connecting them with targeted cargo mitochondria.^44^ In the present study, the mRNA and protein levels of Nur77 and p62 were high and positively correlated with each other in breast tumor tissues. Thus, the regulation of autophagic cell death by Nur77 may be related to autophagic events, such as p62 expression, in breast cancer.

A previous study reported that LNT exhibited antitumor effects in ER^+^ breast cancers.^36^ Moreover, LNT exhibited antitumor activity in MDA‐MB‐231 breast carcinoma cells.^45^ In the present study, compared with those of the control group, the mammary tumors of the LNT‐treated groups were slow‐growing. We preliminarily concluded that LNT can inhibit the growth of mammary tumors in mice and has a preventive function against breast cancer progression. Magnoflorine promotes the anticancer effects of doxorubicin by inducing cellular apoptosis and autophagy in breast cancer cells by regulating the p62, mTOR, and p38 signaling pathways.^31^ Therefore, natural products exert their antitumor effects by regulating autophagic events. Neurensin‐2 (NRSN2) also promotes breast cancer metastasis by activating the PI3K/AKT/mTOR and NF‐κB signaling pathways,^46^ and melittin suppresses EGF‐induced cell motility and invasion by inhibiting the PI3K/Akt/mTOR signaling pathway in breast cancer cells.^47^ This is the first study to confirm that LNT induced autophagic cell death and the NFκB signaling pathway and inhibited the expression of Nur77 and p62. Moreover, LNT could promote autophagic cell death of breast cancer cells by inhibiting the AKT/mTOR and NF‐κB signaling pathways, thus inhibiting the occurrence and development of breast cancer.

The polarization of macrophages contributes to the progression of tumor growth and metastasis.^12^, ^48^, ^49^ One of the hallmarks of malignancy is the polarization of TAMs from a pro‐immune (M1‐like) to an immunosuppressive (M2‐like) phenotype.^50^ An increase in the number of M2‐type macrophages is a marker for poor prognosis and metastasis in various cancer types.^51^, ^52^ TAMs are the key determinants of breast cancer metastasis and progression.^53^ In murine breast cancer models, Oghumu et al.^54^ and Na et al.^55^ confirmed that chemokine (C‐X‐C motif) receptor 3 deficiency enhances tumor progression by promoting macrophage M2 polarization, and the inhibition of cyclooxygenase‐2 expression blocks M2 macrophage differentiation to suppress metastasis. In the peripheral blood, M2‐like monocyte levels are elevated in breast cancer patients compared with those in normal controls and patients with benign breast disease.^56^ Therefore, macrophage polarization plays a key role in breast cancer progression. In the present study, we discovered LNT‐mediated inhibition of breast cancer by monitoring macrophage polarization in mice. Mechanistically, LNT treatment promoted M1 polarization of macrophages in the presence of breast tumor cells; that is, the effects of LNT on macrophage polarization depended on breast cancer cells. Meanwhile, M1 macrophages exerted antitumor and pro‐autophagic effects on cells, which also relied on the secretors of tumor cells in the presence of LNT. Moreover, LNT‐mediated autophagic cell death was dependent on Nur77 expression, which were in accordance with the positive correlation between Nur77 and p62 in human breast tumor tissues. Thus, LNT can control cancer cells in the TME and the function of TAMs by regulating Nur77 expression.

## 5 CONCLUSIONS

**Figure 6:**
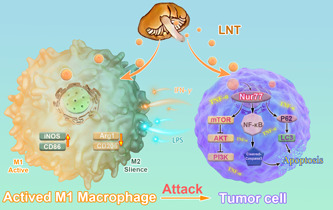
Schematic diagram of *Lentinus edodes* (LNT)‐mediated improvement in the progression of breast cancer. LNT promoted the secretion of cytokines via the regulation of Nur77 and the downstream mTOR/AKT and p62/LC3‐mediated autophagy. The cytokines subsequently inhibited macrophage M2 polarization, which inhibited the AKT/mTOR and NF‐κB signaling axes in breast cancer cells, thereby promoting autophagic tumor cell death and inhibiting breast cancer progression.

LNT inhibited breast tumor growth and restrained the metastasis of breast tumors to the lung tissue in vivo. LNT inhibited the transformation of M1 macrophages into M2 macrophages in tumor tissues and promoted autophagic cell death in breast cancer cells both in vivo and in vitro. Our results provide novel insights into the mechanisms underlying LNT‐induced tumor suppression (Figure 6). Glucans from *L. edodes* or other organisms that produce glucans could be explored as therapeutic options for lung metastasis of breast cancer. Our study revealed that LNT may promote autophagic cell death in breast cancer cells and inhibit the occurrence and progression of breast cancer. However, a large sample size is required to confirm these findings. The role of Nur77 in LNT‐mediated autophagic cell death should be validated using a mouse model. Moreover, the role of Nur77 in LNT‐mediated M1 macrophage polarization should be elucidated in future studies.

## AUTHOR CONTRIBUTIONS

**Fukai Zhu**: Data curation; formal analysis; investigation; methodology; project administration; resources; software; visualization; writing—review and editing. **Qianru Zhang**: Data curation; formal analysis; investigation; methodology; software; visualization. **Jiexin Feng**: Methodology; software; validation. **Xiuru Zhang**: Formal analysis; methodology; software. **Tingting Li**: Formal analysis; software; supervision; validation. **Shuwen Liu**: Formal analysis; methodology; software. **Yanling Chen**: Formal analysis; software; validation. **Xiumin Li**: Data curation; methodology; software. **Qici Wu**: Data curation; formal analysis; methodology. **Yu Xue**: Formal analysis; methodology; resources. **Gulimiran Alitongbieke**: Conceptualization; data curation; formal analysis; funding acquisition; methodology; project administration; resources; software; supervision; validation; writing—original draft. **Yutian Pan**: Conceptualization; project administration; validation; visualization; writing—original draft; writing—review and editing.

## CONFLICT OF INTEREST STATEMENT

The authors declare no conflict of interest.

## ETHICS STATEMENT

All animal studies were approved according to institutional guidelines for laboratory animals. All experiments were approved by the ethics committee of Minnan Normal University (AEWC‐2021010).

## Supporting information


**Figure S1**.

**Figure S2**.

**Figure S3**.

## Data Availability

All data generated or analyzed during this study are included in this published article [and its supplementary information files].
