# Supplementary figures and images for: β‐Glucan produced by Lentinus edodes suppresses breast cancer progression via the inhibition of macrophage M2 polarization by integrating autophagy and inflammatory signals
Source: Immun Inflamm Dis. 2023 May 26;11(5):e876. doi: 10.1002/iid3.876 (PMC10214582; doi:10.1002/iid3.876)

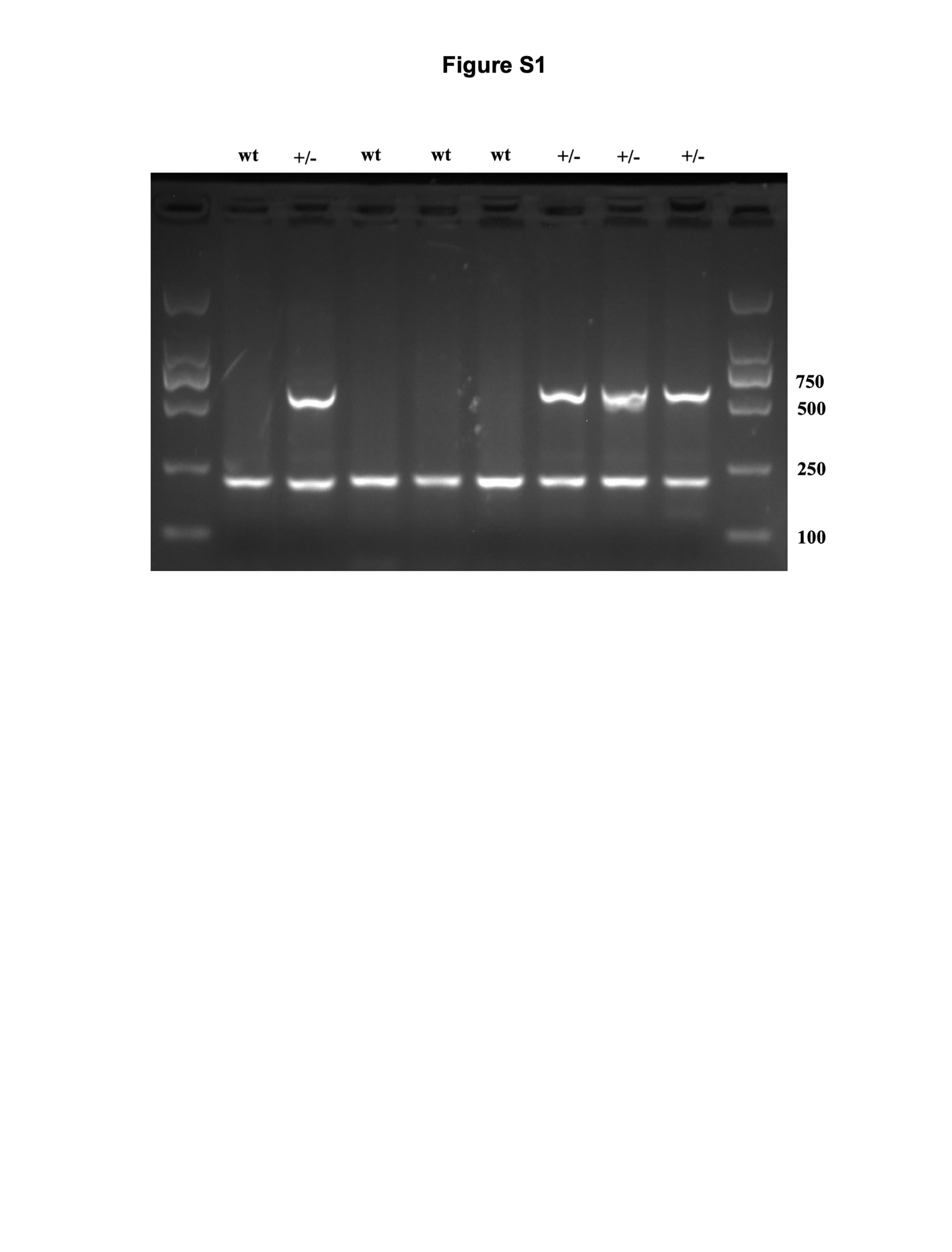

Supplement: Supplementary file 1 — Figure S1. [file IID3-11-e876-s002.jpeg]

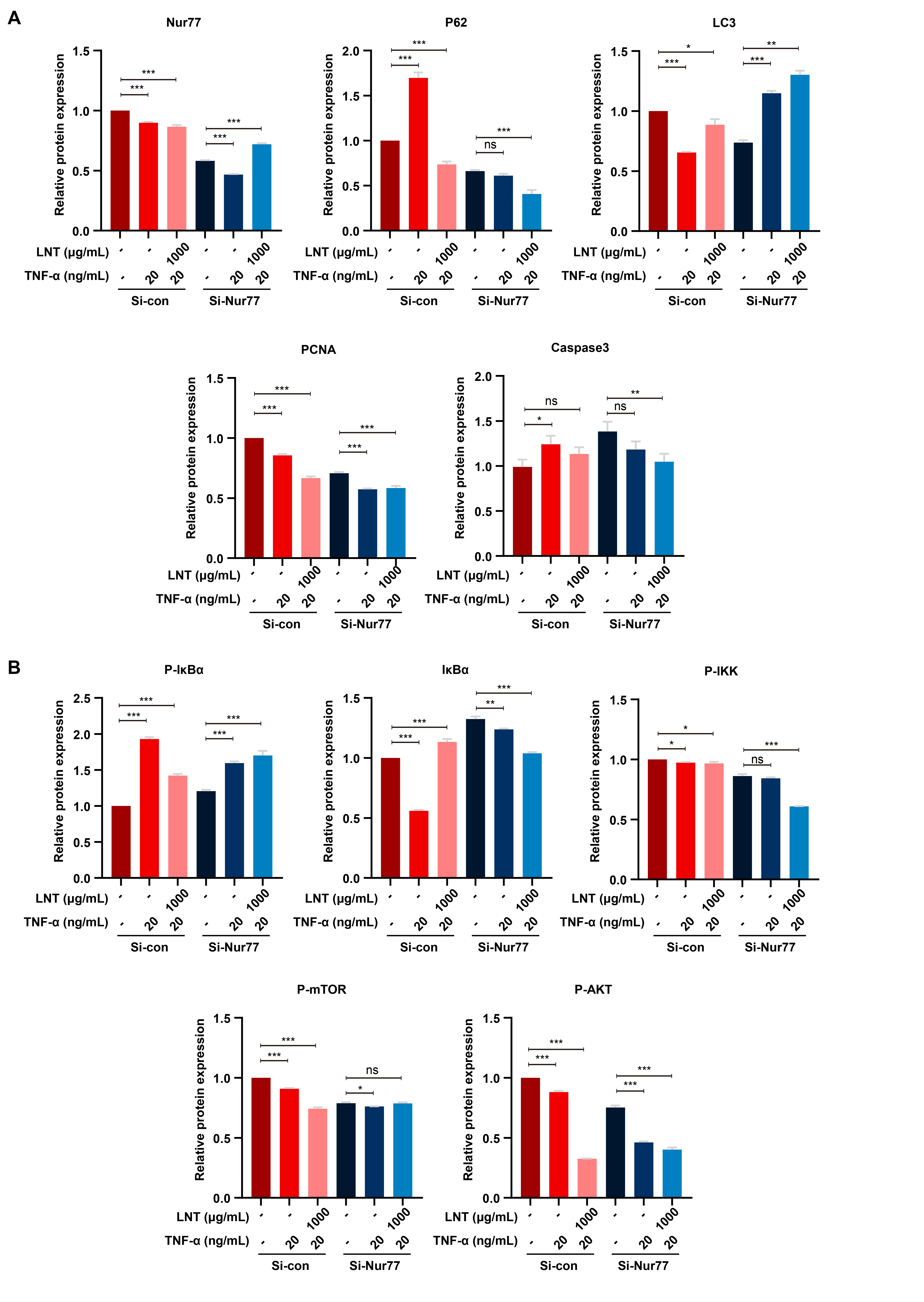

Supplement: Supplementary file 2 — Figure S2. [file IID3-11-e876-s003.jpeg]

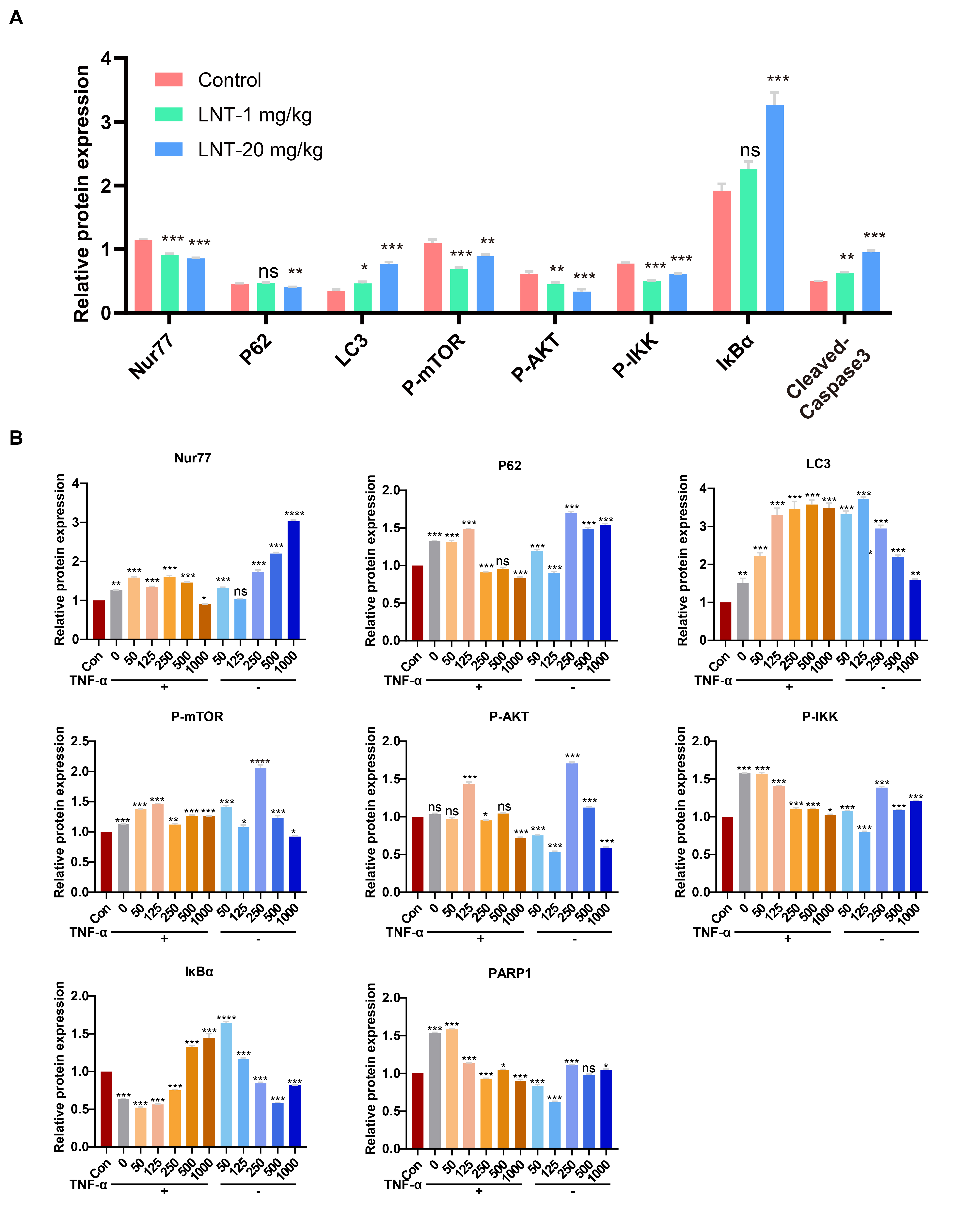

Supplement: Supplementary file 3 — Figure S3. [file IID3-11-e876-s001.jpeg]
